# Exposure to organophosphate insecticides, inappropriate personal protective equipment use, and cognitive performance among pesticide applicators

**DOI:** 10.3389/fpubh.2022.1060284

**Published:** 2022-11-17

**Authors:** Jiraporn Chittrakul, Ratana Sapbamrer, Surat Hongsibsong

**Affiliations:** ^1^Department of Community Medicine, Faculty of Medicine, Chiang Mai University, Chiang Mai, Thailand; ^2^School of Health Sciences Research, Research Institute for Health Sciences, Chiang Mai University, Chiang Mai, Thailand

**Keywords:** organophosphate, insecticide, pesticide, personal protective equipment, cognitive performance

## Abstract

Inappropriate use of personal protective equipment (PPE) among pesticide applicators may increase urinary organophosphate (OP) metabolite levels and subsequently increase risks of cognitive performance. Therefore, this study aims to (1) compare urinary OP metabolite levels and cognitive performance between pre-and post-pesticide application seasons; (2) PPE use and factors associated with PPE use linked to increased urinary OP metabolite levels during pesticide application; and (3) the association between urinary OP metabolite levels and cognitive performance. This longitudinal follow-up study on 79 pesticide applicators was carried out between October 2021 and January 2022. The applicators were interviewed, collected urine samples, and tested for cognitive performance in pre-and post-pesticide application seasons. The results found that the levels of urinary OP metabolites in post-application season were significantly higher than those in pre-application season (*p* < 0.001). Multiple linear regression analysis found that increased total diethylphosphate (DEP) and total dialkylphosphate (DAP) levels were associated with not wearing gloves while mixing pesticides [beta (β) ± standard error (SE) = −43.74 ± 18.52, 95% confidence interval (95% CI) = −80.84, −6.64 for total DEP and −50.84 ± 19.26, 95% CI = −89.41, −12.26 for total DAP] and also with not wearing a mask while spraying pesticides (β ± SE = −31.76 ± 12.24, 95% CI = −56.28, −7.24 for total DEP and −33.20 ± 12.63, 95% CI = −58.49, −7.92 for total DAP) after adjusting for covariates. The scores of Montreal Cognitive Assessment-Thai, Thai Mental State Examination, and Mini-Cognitive test in post-pesticide application were significantly lower than those in pre-pesticide application (*p* < 0.001). However, no association was found between urinary OP metabolite levels and cognitive decline. Our findings indicate that inappropriate PPE use during pesticide application was the major factor affecting urinary OP metabolite levels among pesticide applicators. Wearing gloves when mixing pesticides and a mask when spraying pesticides were key factors in reducing occupational exposure to OP. Exposure to OP at low levels and for short periods of exposure may not affect cognitive performance significantly. Therefore, long-term exposure and exposure to high levels of OP should be investigated further.

## Introduction

Pesticides are widely and extensively used worldwide in agriculture for controlling pests such as insects, rodents, and disease organisms (1). Insecticides are most frequently used in agriculture and the one of most common groups used insecticides is organophosphates (OP) (2, 3). Farmers and agricultural workers are at high risk of being exposed to these chemicals, the main routes of exposure being dermal contact, ingestion, and inhalation (4, 5). Exposure to these chemicals can cause both acute and chronic health consequences (4). Several studies have shown that exposure to OP was associated with chronic diseases such as asthma, rheumatoid arthritis, cancers, Alzheimer's disease, Parkinson's disease, and cognitive impairment (6–9).

The main mechanism contributing to the OP toxicity is that they inhibit red blood cells and the enzyme plasma cholinesterase (AChE) in the nervous system, which is responsible for the degradation of acetylcholine. When inhibition of AChE occurs, acetylcholine builds up in the neuro muscular and synaptic gaps, leading to an overabundance of acetylcholine within them (10). Therefore, neurological health problems demonstrate the main effects of OP toxicity. Furthermore, OP may have an effect on cognitive performance through cholinergic stimulation, oxidative stress, inflammation induction, and mitochondrial dysfunction in the nervous system (11, 12). Previously available studies found that long-term exposure to OP was associated with neurological disorders such as Alzheimer's disease, dementia, Parkinson's disease, and cognitive impairment (8, 9). Mild cognitive impairment is an early stage of cognitive impairment that leads to Alzheimer's disease and dementia. A study by Kim et al. (13) investigated the relationship between pesticide exposure and cognitive decline in a rural South Korean population, and they found that exposure to pesticides was associated with cognitive impairment. A study by Ramirez-Santana et al. (14) also investigated the effect of chronic exposure to OP on the neurobehavioral performance among agricultural workers and rural inhabitants, and they suggested that occupational exposure to OP resulted in a significantly lower neurobehavioral performance. Exposure to OP in farmworkers had also an effect on executive function, language, memory, attention, processing speed, visual-spatial function, and coordination (15). However, previously available studies had limitations such as those associated with study design, indirect measurement of OP exposure (questionnaire or measurement of AChE inhibition) (13–15). Although there is evidence that low-level exposure to OP were linked with cognitive decline and impaired neurobehavioral function, the potential effects of long-term low-level exposure to OP are unclear (16, 17).

In Thailand, agriculture employs more than 30% of the workforce and accounts for a major economic sector of the gross domestic product of the country. Pesticide use has risen over the past decade, and OP are some of the most used insecticides for controlling pests in agriculture (18, 19). Thai farmers are at high risk of adverse health effects from pesticide exposure due to lack of pesticide knowledge, unsafe occupational practices, and improper use of PPE (19). In this study, we hypothesized that improper use of PPE among pesticide applicators may increase urinary OP levels, and subsequently increase risk of cognitive impairment. Therefore, this study aims to: (a) compare urinary OP metabolite levels and cognitive performance between pre-and post-pesticide application seasons; (b) PPE use, and factors of PPE use associated with increased urinary OP metabolite levels during pesticide application; and (c) the association between urinary OP metabolite levels and cognitive performance.

## Materials and methods

### Study population

This study was carried out in an agricultural area in the Phrao and Samoeng Districts of Chiang Mai province, northern Thailand. This area plants potatoes every year during November and January. The major pesticides used in potato fields were OP insecticides. Therefore, this study was conducted from October 2021 to January 2022. All farmers prepared to start a new crop of potatoes in October 2021, and they had not been exposed to any pesticides in the previous 1–2 months. They began planting the new crop in November 2021, and applied OPs to their fields during December 2021 and January 2022. Eligibility criteria were pesticide applicators over the age of 18 years who were healthy without current illness, planted potatoes between October 2021 and January 2022, and sprayed OP insecticides on their crops. The applicators who had neurological and psychological problems were ineligible to participate in the study.

Out of 128 applicators in the study area, 104 (81.3%) fulfilled the eligibility criteria, 93 (89.4%) agreed to participate in the study, 14 (15.1%) dropped out during the follow-up period. Seventy-nine applicators therefore were the study subjects with a response rate of 76%.

### Study design

This study was a longitudinal follow-up study on 79 pesticide applicators to compare urinary OP metabolite levels and cognitive performance between pre-and post-pesticide application seasons. The study also investigated PPE use and factors of PPE use associated with increased urinary OP metabolite levels during pesticide application. The applicators were interviewed, collected urine samples, and were tested for cognitive performance twice, once in each of the pre-and post-pesticide application seasons. Pre-pesticide application season samples were collected in October 2021, and post-pesticide application season samples were collected in January 2022. The period between pre-and post-pesticide application was about 2–3 months.

### Interviews

All subjects were interviewed face-to-face for approximately 15 min by trained interviewers. The questions in the validated interview form were in 3 parts: (1) demographic data (age, gender, marital status, education level, monthly income, underlying disease, body mass index, smoking and alcohol consumption); (2) information regarding pesticide use (frequency of pesticide use, duration of pesticide spraying, type of pesticide use, type of sprayer equipment, storage of pesticide containers and equipment, method of pesticide container disposal, distance from farm to residence, information about pesticides); and (3) PPE use when mixing pesticides, spraying pesticides, and cleaning spraying equipment. The PPE checklists included hat, goggles, long-sleeve shirt, long-sleeve trousers, boots, mask, and gloves. The subjects were interviewed with regard to demographic data in the pre-pesticide application season, the information with regard to pesticides and PPE use being collected in the post-pesticide application season.

### Analysis of urinary OP metabolites

Three hundred mL of spot morning urine samples were collected twice, once in each of the pre-and post-pesticide application seasons to determine urinary OP metabolite levels. The urine samples were collected in polypropylene containers and stored at 2–3 °C in cooler boxes with ice until transferred to the laboratory of the Research Institute for Health Sciences, Chiang Mai University within a day after collection. The urine sample was then aliquoted into 12 mL portions and stored in a freezer at −20 °C until analysis within a month after collection. The OP metabolites were extracted and analyzed in accordance with the method described by Prapamontol et al. (20). Six OP metabolites were analyzed, including diethylphosphate (DEP), diethylthiophosphate (DETP), diethyldithiophosphate (DEDTP) dimethylphosphate (DMP) dimethylthiophosphate (DMTP), and dimethyldithiophosphate (DMDTP). Total DEP represented the summation of DEP, DETP, and DEDTP. Total DMP represented the summation of DMP, DMTP, and DMDTP. Total DAP represented the summation of total DEP and total DMP. The OP metabolite levels were calculated and presented as microgram/ gram creatinine (μg/g creatinine).

To ensure quality control, the limit of detection (LOD) ranged from 0.1 μg/L for DETP to 1.0 μg/L for DMP, whereas the limit of quantification (LOQ) ranged from 0.4 μg/L for DETP to 3.5 μg/L for DMP. Recovery ranged from 82.0% for DMDTP to 114.5% for DEP. The coefficient of variation (%CV) of intra-batch ranged from 3.9% for DMP to 7.3% for DMDTP, and the %CV of inter-batch ranged from 4.07% for DMP to 7.92% for DEP. The concentrations below LOD were replaced with LOD divided by the square root of 2 (21).

### Test of cognitive performance

Cognitive performance was assessed by certified physical therapists, a process taking 25–30 min. Assessment was carried out in pre-and post-pesticide application seasons. The tests used for assessment of cognitive performance included the Montreal Cognitive Assessment - Thai version (MoCA-T), the Thai Mental State Examination (TMSE), and the Mini-Cognitive Test (Mini-Cog). These tests are widely recognized cognitive performance screening tools.

The MoCA-T is a tool for detecting cognitive performance by using a paper-and-pencil tool. The MoCA-T assesses multiple cognitive domains including attention, concentration, executive functions, memory, language, visuospatial skills, abstraction, calculation, and orientation with a maximum score of 30. The reliability (Cronbach's alpha coefficient) of the MoCA-T is 0.80 (22). The TMSE is a tool for screening cognitive performance by assessing orientation, registration, attention, calculation, recall, language, repetit3-stage command, reading, writing, and copying with a maximum score of 30. The sensitivity and specificity of the TMSE are 82 and 70%, respectively (23). The Mini-Cog Test is a short form of a brain performance assessment for detection of cognitive performance. There are three questions to assess cognition and memory with a maximum score of 3. The sensitivity and specificity of the Mini-Cog test are 72.8 and 97.6% (24, 25).

### Data analysis

Descriptive statistics are presented as frequency (n), percentage, (%), mean, geometric mean (GM), median, standard deviation (SD), standard error (SE), the 25^th^ percentile (P^25th^), and the 75^th^ percentile (P^75th^). All parameters were tested for normality of distribution by using Komolgorov-Smirnov and Shapiro-Wilk tests. The Mann-Whitney U Test was used to determine the difference in urinary OP metabolite levels and scores of cognitive performance between pre-and post-pesticide application seasons as these data did not show a normal distribution. The Mann–Whitney *U* and Kruskal Wallis tests were used to determine the change in urinary OP metabolite levels classified by demographic data and agricultural information. Multiple linear regression was used to analyze the impact of factors associated with PPE use on increased urinary OP metabolite levels. The enter method was used for analysis, and the potential covariates of increased urinary OP metabolite levels (*p* value < 0.20) were included in the model of the multiple linear regression analysis. These data are presented as beta (β), standard error (SE), 95% confidence interval (95% CI). The Spearman rank correlation coefficient (*r*) was calculated and used to determine the correlation between the increase in urinary OP metabolite levels and cognitive decline. A *p* value of < 0.05, was considered as statistically significant.

### Ethical approval

The study was approved by the Research Ethics Committee, Faculty of Medicine, Chiang Mai University (no. 219/2021). All subjects provided written informed consent before participating in the study.

## Results

### Demographic characteristics and agricultural information of the pesticide applicators

Seventy-nine applicators (*n* = 79) participated in this study. The mean age of the applicators was 52.9 ± 10.2 years. The majority of the participants were male (77.2%), educated to primary school level or below (70.9%), married (87.3%), and had a monthly income ≤ 10,000 Thai Baht (89.9%). Only 17.7% of the applicators smoked cigarettes, while 40.5% consumed alcohol. With regard to farm experience, most farmers (83.5%) had applied pesticides for longer than 10 years, had applied pesticides approximately 1–4 days per week (81.0%), and sprayed pesticides for longer than 2 h per day (67.1%). The most commonly used OP in this study area are triazophos (88.6%), followed by chlorpyrifos (15.2%). As regards spraying equipment, the majority of the applicators used motorized tank sprayers for spraying pesticides (69.6%), followed by motorized knapsack sprayers (17.7%), and hand knapsack sprayers (12.7%). Around 63.3% of the applicators stored pesticide containers and equipment at home, and 35.4% disposed of the pesticide containers in landfill. The majority of applicators (87.3%) lived between 1 and 5 kilometers from the farm (Table 1).

**Table 1:** Demographic characteristics and agricultural information of pesticide applicators (*n* = 79).

**Variable**		***n* (%) or mean±SD**
Age (years)		52.9 ± 10.2
Gender	Male	61 (77.2)
	Female	18 (22.8)
Marital status	Single/divorced	10 (12.7)
	Married	69 (87.3)
Education level	Primary school or lower	56 (70.9)
	Secondary school	20 (25.3)
	Bachelor's degree or higher	3 (3.8)
Monthly income	≤ 10,000 Thai Baht	71 (89.9)
	>10,000 Thai Baht	8 (10.1)
Underlying disease		27(34.2)
Body mass index (BMI) (kg/m^2^)		23.4 ± 4.1
Smoker of cigarettes		14(17.7)
Alcohol consumption		32(40.5)
Duration of pesticide use (years)	1–10 years	13 (16.5)
	>10 years	66 (83.5)
Frequency of pesticide use	1–4 days/week	64 (81.0)
	>4 days/week	15 (19.0)
Duration of pesticide spraying	0–2 h	26 (32.9)
	>2 h	53 (67.1)
Type of pesticide use	Triazophos	70 (88.6)
	Chlorpyrifos	12 (15.2)
	Diazinon	1 (1.3)
	Glyphosate	73 (92.4)
	Paraquat	27 (34.2)
Type of spraying equipment	Hand knapsack sprayer	10 (12.7)
	Motorized knapsack sprayer	14 (17.7)
	Motorized tank sprayer	55 (69.6)
Type of spray nozzle	Low pressure	63 (79.7)
	High pressure	16 (20.3)
Storage of pesticide containers and equipment	Home	50 (63.3)
	field	29 (36.7)
Method of pesticide container disposal	Landfill	28 (35.4)
	Burn	12 (15.2)
	Reuse	28 (35.4)
	Sell	11 (13.9)
Distance from farm to residence	1–5 kilometers	69 (87.3)
	>5 kilometers	10 (12.7)
Have information about pesticides	Yes	45 (57.0)
	No	34 (43.0)

### Urinary OP metabolite levels between pre-and post-pesticide application seasons

Table 2 shows the levels of six OP metabolites in pre-and post-pesticide application seasons. The percentage of OP metabolites detected in pre-pesticide application ranged from 3.9% for DEDTP and DMP to 51.9% for DEP, while the % detected in the post-pesticide application season ranged from 1.3% for DMP and DMDTP to 73.4% for DEP.

**Table 2:** Urinary OP metabolite levels (μg/g creatinine) between pre-and post-pesticide application seasons (*n* = 79).

**Metabolite**	**Pre-pesticide application season**			**Post-pesticide application season**			***p* value**
	**% detected**	**GM (SE)**	**Median (P^25th^, P^75th^)**	**% detected**	**GM (SE)**	**Median (P^25th^, P^75th^)**	
DEP	51.9	0.56 (0.43)	0.41 (0.23,1.29)	73.4	3.14 (3.21)	3.31 (0.85,8.97)	< 0.001^**^
DETP	21.5	0.13 (0.05)	0.10 (0.07,0.23)	45.6	0.57 (1.06)	0.31 (0.13, 2.57)	< 0.001^**^
DEDTP	3.8	0.12 (0.16)	0.12 (0.08,0.19)	2.5	0.07 (0.01)	0.08 (0.05, 0.12)	< 0.001^**^
DMP	3.8	0.97 (1.25)	0.86(0.54,1.28)	1.3	1.46 (0.19)	1.40 (0.93, 2.13)	< 0.001^**^
DMTP	11.4	0.34 (0.23)	0.28 (0.17, 0.45)	3.8	0.46 (0.09)	0.63(0.30, 0.66)	< 0.001^**^
DMDTP	2.5	0.18 (0.03)	0.17 (0.11, 0.25)	1.3	0.28 (0.03)	0.26 (0.18, 0.41)	< 0.001^**^
Total DEP	51.9	1.07(0.47)	0.63 (0.46,2.07)	73.4	4.43 (3.91)	4.18 (1.17, 12.41)	< 0.001^**^
Total DMP	11.4	1.61 (1.28)	1.38 (0.83,2.14)	3.8	2.22 (0.30)	2.11 (1.47,3.20)	< 0.001^**^
Total DAP	60.8	3.25 (1.35)	2.51 (1.74, 5.39)	73.4	8.67 (3.95)	18.54 (3.97, 15.43)	< 0.001^**^

DEP, diethylphosphate; DETP, diethylthiophosphate; DEDTP, diethyldithiophosphate; DMP, dimethylphosphate; DMTP, dimethylthiophosphate; DMDTP, dimethyldithiophosphate; DAP, dialkylphosphate; Total DEP, summation of DEP, DETP, and DEDTP; Total DMP, summation of DMP, DMTP, and DMDTP; Total DAP, summation of total DEP and total DMP; GM, geometric mean; SE, standard error; P^25th^, the 25^th^ percentile; P^75th^, the 75^th^ percentile;

** *p* value < 0.001.

Total DAP levels in post-pesticide application (GM ± SE = 8.67 ± 3.95 μg/g creatinine) were significantly higher than those in the pre-pesticide application samples (GM ± SE = 3.25 ± 1.35 μg/g creatinine). Similarly, total DEP and total DMP levels in the post-pesticide application samples (GM ± SE = 4.43 ± 3.91 μg/g creatinine for total DEP and 2.22 ± 0.30 μg/g creatinine for total DMP) were significantly higher than those pre-pesticide application (GM ± SE = 1.07 ± 0.47 μg/g creatinine for total DEP and 1.61 ± 1.28 μg/g creatinine for total DMP).

Changes in urinary OP metabolite levels during pesticide application classified by demographic characteristics and agricultural information are shown in Table 3. The applicators who used motorized tank sprayers had higher total DEP levels than the applicators who used motorized knapsack sprayers (*p* value = 0.016).

**Table 3:** Change in urinary OP metabolite levels during pesticide application classified by demographic characteristics and agricultural information.

**Parameter**		Δ **total DEP**		Δ **total DMP**		Δ **total DAP**	
		**Median (P^25th^, P^75th^)**	***p* value**	**Median (P^25th^, P^75th^)**	***p* value**	**Median (P^25th^, P^75th^)**	***p* value**
Gender	Male	1.65 (0.21,9.25)	0.112	0.60 (−0.05,1.28)	0.653	2.78 (0.32, 11.99)	0.124
	Female	7.87 (0.93, 19.52)		0.76 (−0.10, 2.73)		6.39 (2.97, 22.34)	
Marital status	Single/divorced	2.18 (−1.13, 23.74)	0.573	0.32 (−5.35, 1.05)	0.423	3.29 (−6.14, 18.89)	0.43
	Married	2.39 (0.34, 10.75)		0.67 (−6.14, 1.61)		4.12 (1.05, 11.99)	
Education level	Primary school or lower	2.79 (0.23, 19.94)	0.474	0.63 (−0.85, 1.43)	0.058	3.82 (0.51, 17.82)	0.640
	Secondary school	1.34 (0.32,6.30)		0.29 (−0.06, 1.16)		2.43 (0.48, 7.81)	
	Bachelor's degree or higher	0.87 (0.72, 0.87)		3.48(2.57, 3.48		4.35 (3.29, 4.35)	
Monthly income	≤ 10,000 Thai Baht	2.18 (0.22, 11.80)	0.697	0.63 (−0.06,1.59)	0.371	3.51 (0.76, 12.84)	0.363
	>10,000 Thai Baht	2.56 (0.99, 7.78)		0.14 (−10.68, 1.33)		2.88 (−8.73, 7.25)	
Underlying disease	Yes	2.18 (0.27, 10.22)	0.914	0.84 (0.23, 1.45	0.158	4.35 (1.33, 12.84)	0.591
	No	2.29 (0.33, 11.57)		0.47 (−0.85, 1.58)		3.33 (0.51, 9.15)	
Smoker of cigarettes	Yes	1.11 (−0.73,7.37)	0.186	0.27 (−0.780.89	0.227	1.24 (−0.34, 8.77)	0.136
	No	2.49 (0.42, 11.35)		0.68 (−0.07, 1.64)		4.35 (1.09, 12.75)	
Alcohol consumption	Yes	2.5 (0.65, 9.73)	0.621	0.63 (−0.15, 1.61)	0.924	3.64 (1.20, 12.32)	0.822
	No	1.61 (0.19, 12.24)		0.60 (−0.07, 1.45)		3.51 (0.29, 12.84)	
Duration of pesticide use	1–10 years	1.07 (0.80, 15.00)	0.884	0.83 (0.14, 3.18)	0.175	4.61 (2.17, 13.51)	0.561
	>10 years	2.29 (0.26, 11.12)		0.57 (−0.13, 1.40)		3.09 (0.40, 12.70)	
Frequency of pesticide use	1–4 days/week	2.02 (0.28,10.82)	0.955	0.61 (−0.07, 1.56)	0.871	3.81 (0.97, 12.32)	0.832
	>4 days/week	2.91 (0.27, 11.80)		0.68 (−0.71, 1.54)		2.78 (0.07, 17.34)	
Duration of pesticide spraying	0–2 h	2.58 (0.50,8.81)	0.950	0.91 (0.31, 1.76)	0.071	4.85 (1.35, 11.71)	0.549
	>2 h	1.85 (0.25, 14.20)		0.33 (−0.80, 1.57)		3.02 (0.32, 14.94)	
Type of spraying equipment	Hand knapsack sprayer	1.09 (−0.41,6.72)	0.016^*^^ab^	0.57 (−0.04, 1.10)	0.370	0.77 (−0.72, 5.27)	0.060
	Motorized knapsack sprayer ^a^	0.65 (0.15, 1.67)		0.99 (0.19, 3.23)		2.75 (1.07, 4.57)	
	Motorized tank sprayer ^b^	3.49 (0.58, 19.40)		0.54 (−0.71, 1.54)		5.41 (1.04, 19.80)	
Type of spray nozzle	Low pressure	2.18 (0.19, 10.89)	0.329	0.63 (−0.02, 1.38)	0.860	3.16 (0.29, 12.8)	0.209
	High pressure	2.30 (0.78, 11.50)		0.16 (−0.72, 2.57)		4.93 (2.19, 9.15)	
Storage of pesticide containers and equipment	Home	2.18 (0.25, 8.29)	0.138	0.37 (−0.13, 1.56)	0.476	2.61 (0.29, 8.53)	0.431
	Field	3.49 (0.40, 18.03)		0.68 (0.24, 1.37)		4.55 (1.62, 18.90)	
Method of pesticide container disposal	Landfill	2.34 (0.75,10.03)	0.629	0.28 (−1.80, 1.58)	0.685	3.82 (1.37, 9.15)	0.557
	Burn	2.09 (0.67, 22.69)		0.53 (0.30, 2.21)		4.69 (1.84, 23.01)	
	Reuse	1.32 (−0.06, 11.22)		0.68 (−0.56, 1.43)		1.98 (0.16, 11.82)	
	Sell	4.54 (0.11, 19.40)		0.68 (−0.06, 2.27)		5.41 (0.34, 22.27)	
Distance from farm to residence	1–5 kilometers	2.39 (0.30,10.75)	0.779	0.68 (−0.08, 1.61)	0.140	4.11 (0.86, 11.99)	0.555
	>5 kilometers	0.68 (−0.29, 19.29)		0.14 (−0.38, 0.62)		1.96 (−0.74, 17.90)	
Have information about pesticides	Yes	1.61 (0.21,14.20)	0.579	0.62 (−0.08, 1.50)	0.886	3.16 (0.36, 14.94)	0.681
	No	2.79 (0.53, 10.32)		0.64 (−0.08, 1.64)		4.69 (1.02, 11.71)	

Δ total DEP, total DEP in post-pesticide application - total DEP in pre-pesticide application; Δ total DMP, total DMP in post-pesticide application - total DMP in pre-pesticide application; Δ total DAP, total DAP in post-pesticide application - total DAP in pre-pesticide application; P^25th^, the 25^th^ percentile; P^75th^, the 75^th^ percentile;

* *p* value < 0.05;

ab the applicators who used motorized tank sprayers had higher total DEP levels than the applicators who used motorized knapsack sprayers.

### PPE use while mixing pesticides, spraying pesticides, and cleaning spraying equipment

**Figure 1:**
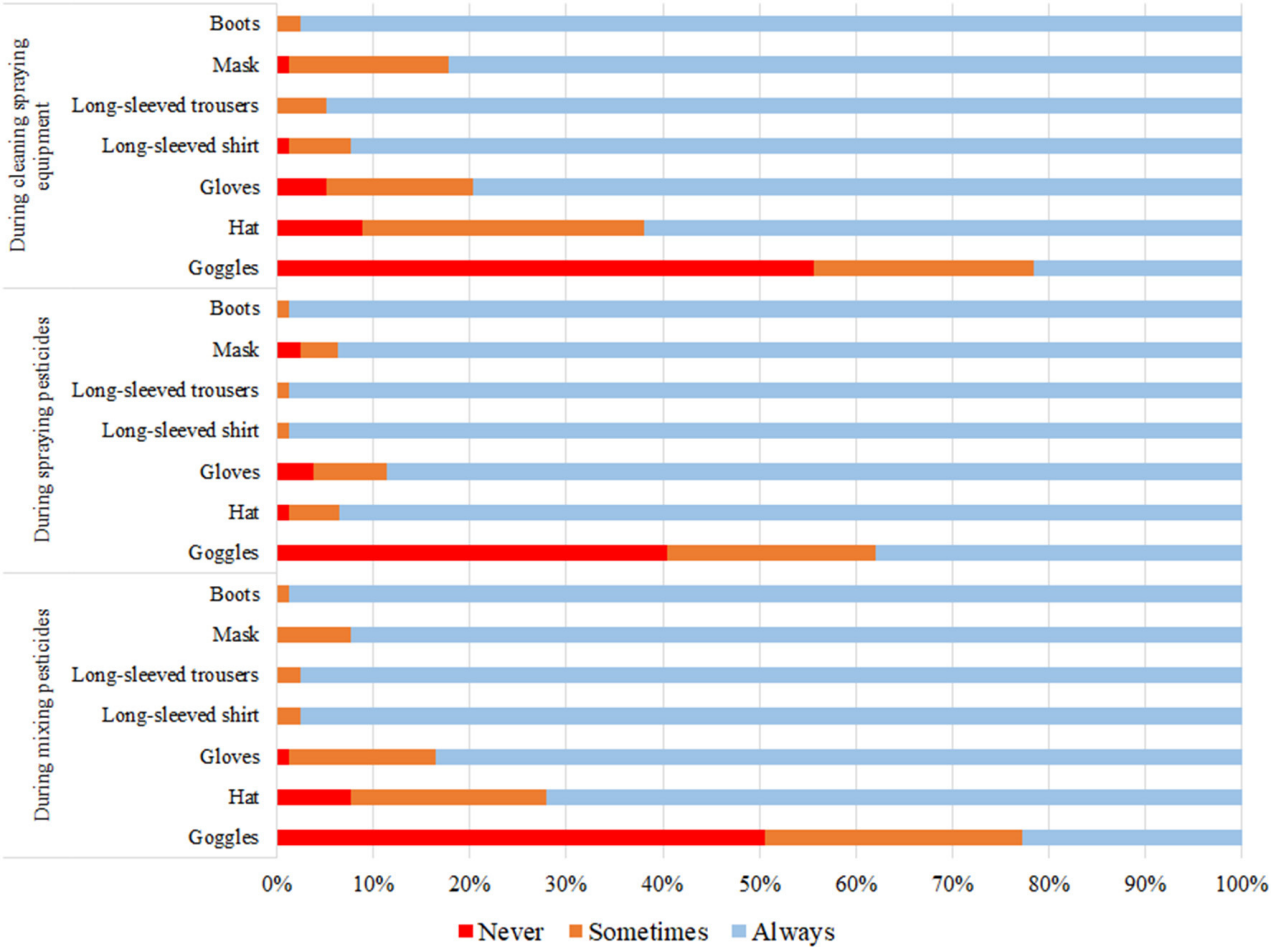
PPE use during pesticide application.

While mixing pesticides, half of the applicators (50.6%) never wore goggles, hat (7.6%), and gloves (1.3%). Most applicators always wore boots, a long-sleeve shirt, and long-sleeve trousers (98.7, 97.5, and 97.5%, respectively). While spraying pesticides, 40.5% never wore goggles, gloves (3.8%), and hat (1.3%). Most applicators always wore boots, a long-sleeve shirt, and long-sleeve trousers (98.7%). While cleaning sprayer equipment, 55.7% never wore goggles, hat (8.9%), and gloves (5.1%). Most applicators always wore boots, long-sleeve trousers, and long-sleeve shirt (97.5, 94.9, and 92.4%, respectively) (Figure 1).

**Figure 2:**
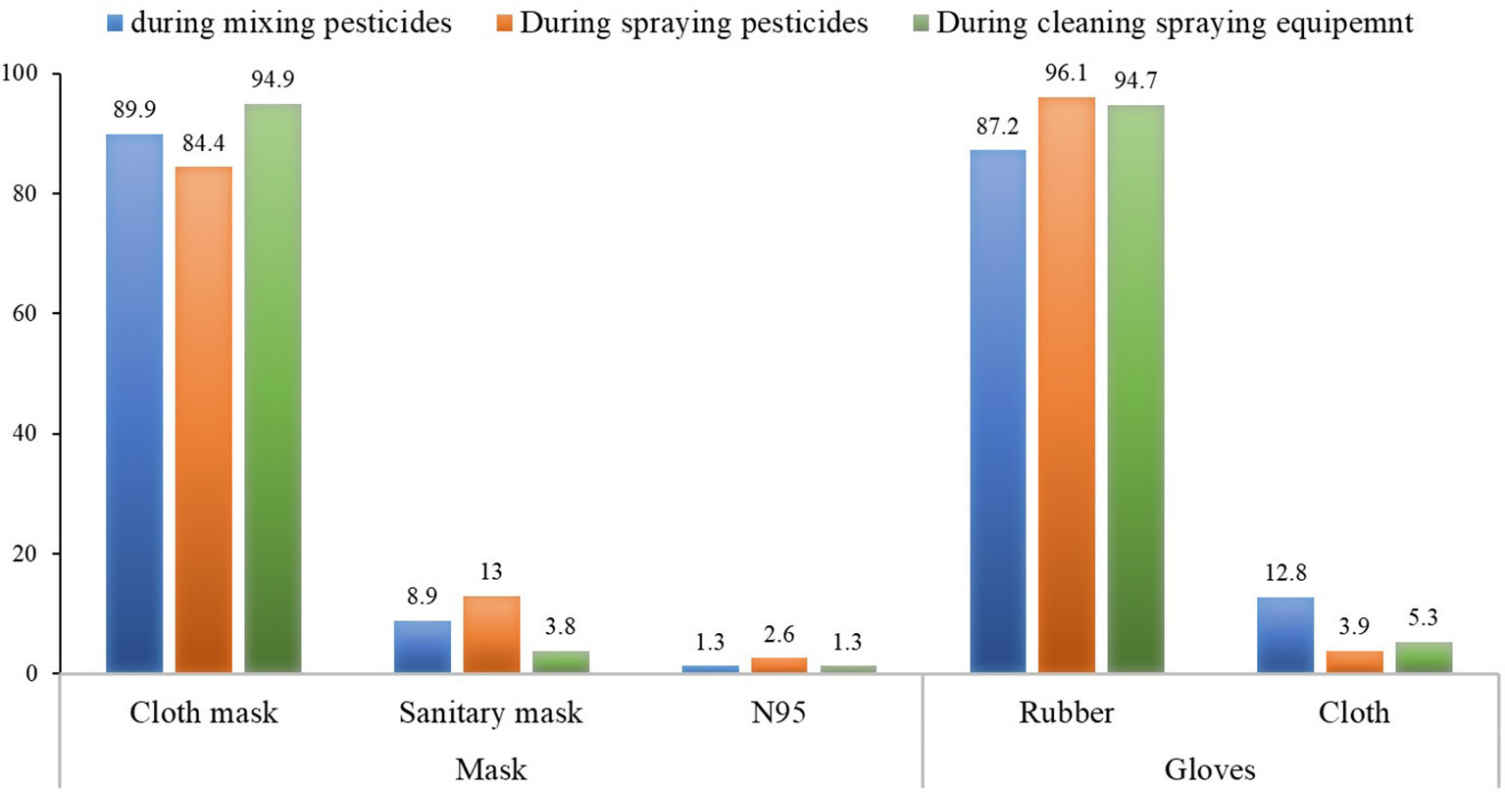
Types of mask and gloves worn during pesticide application.

Regarding the type of mask and gloves worn during pesticide application, the most common mask worn was a cloth mask (84.4–94.9%), followed by a surgical mask (3.8–13%) and N95 (1.3–2.6%), respectively. Most applicators wore rubber gloves (87.2–96.1%), although some wore cloth gloves (3.9–12.8%) (Figure 2).

### Factors of PPE use on increased urinary op metabolite levels during pesticide application

Multiple linear regression analysis indicated that increased urinary total DEP levels were associated with not wearing gloves while mixing pesticides (β ± SE = −43.74 ± 18.52, 95% CI = −80.84, −6.64) and not wearing a mask during spraying pesticides (β ± SE = −31.76 ± 12.24, 95% CI = −56.28, −7.24) after adjusting for covariates. Similarly, total DAP levels were associated with not wearing gloves during mixing pesticides (β ± SE= −50.8 ± 19.26, 95% CI = −89.41, −12.26) and not wearing mask while spraying pesticides (β ± SE = −33.20 ± 12.63, 95% CI = −58.49, −7.92) after adjusting for covariates (Table 4).

**Table 4:** Factors of PPE use on increased urinary OP metabolite levels during pesticide application.

**Parameter**	**Increased total DEP^a^**		**Increased total DMP^b^**		**Increased total DAP^c^**	
	**β ±SE**	**95% CI**	**β ±SE**	**95% CI**	**β ±SE**	**95% CI**
**Wearing PPE during mixing pesticides**						
Hat	−4.77 ± 9.09	−22.98, 13.44	−1.20 ± 3.72	−9.45, 5.46	−7.61 ± 9.50	−26.64, 11.42
Goggles	−10.46 ± 11.70	−33.89, 12.98	0.48 ± 4.60	−8.74, 9.71	−8.38 ± 12.23	−32.87, 16.11
Mask	25.29 + 20.10	−14.98, 65.56	5.53 ± 7.93	−10.37, 21.42	29.77 ± 20.83	−11.94, 71.48
Gloves	**−43.74** **±18.52**	**−80.84**, −6.64*	−7.80 ± 7.26	−22.36, 6.76	**−50.84** **±19.26**	**−89.41**, −12.26*
Long–sleeve trousers	67.32 ± 46.73	−26.29, 160.94	5.36 ± 18.38	−31.49, 42.21	66.67 ± 48.47	−30.39, 163.7
**Wearing PPE during spraying pesticides**						
Hat	21.21 ± 17.23	−13.31, 55.73	3.60 ± 6.76	−9.96, 17.15	24.47 ± 18.01	−11.59, 60.53
9 Goggles	16.17 ± 9.56	−2.99, 35.33	2.16 ± 4.08	−6.02, 10.34	18.02 ± 9.96	−1.92, 37.96
Boots	−93.80 ± 70.33	−234.7, 47.09	−10.54 ± 28.28	−67.23, 46.15	−101.62 ± 73.38	−248.6, 45.32
Mask	**−31.76** **±12.24**	**−56.28**, −7.24*	−2.71 ± 4.72	−12.18, 6.76	**−33.20** **±12.63**	**−58.49**, −7.92*
Gloves	0.161 ± 16.45	−32.78, 33.11	−0.47 ± 6.43	−13.37, 12.43	0.748 ± 17.10	−33.49, 34.99
**Wearing PPE during cleaning sprayer equipment**						
Hat	0.464 ± 8.77	−17.10, 18.03	0.63 ± 3.64	−6.66, 7.93	0.65 ± 9.13	−17.64, 18.94
Goggles	−5.77 ± 8.86	−23.51, 11.98	−1.43 ± 3.47	−8.39, 5.54	−7.32 ± 9.25	−25.84, 11.21
Boots	−16.78 ± 45.73	−108.38, 74.81	2.57 ± 18.02	−33.56, 38.71	−13.49 ± 46.99	−107.6, 80.61
Mask	−0.35 ± 12.76	−25.91, 25.21	−1.44 ± 5.16	−11.78, 8.90	−2.28 ± 13.30	−28.91, 24.35
Gloves	11.49 ± 12.11	−12.76, 35.74	3.25 ± 5.21	−7.19, 13.70	13.57 ± 12.66	−11.77, 38.91
Long–sleeve shirt	5.60 ± 17.07	−28.59, 39.79	2.83 ± 7.0	−11.20, 16.87	7.31 ± 17.82	−28.37, 43.00
Long–sleeve trousers	−6.52 ± 34.13	−74.90, 61.85	−7.89 ± 13.58	−35.12, 19.33	−18.84 ± 35.44	−89.81, 52.14

a Adjusted with age, gender, smoking status, type of spraying equipment, and storage of pesticide containers and equipment.

b Adjusted with age, gender, education level, underlying disease, duration of pesticide use, duration of pesticide spraying, and distance from farm to residence.

c Adjusted with age, gender, smoking status, and type of spraying equipment.

Total DEP, the summation of diethylphosphate (DEP), diethylthiophosphate (DETP), and diethyldithiophosphate (DEDTP).

Total DMP, the summation of dimethylphosphate (DMP), dimethylthiophosphate (DMTP), and dimethyldithiophosphate (DEDTP).

Total DAP, the summation of total DEP and total DMP.

β, beta; SE, standard error; 95% CI, 95% confidence interval; *p value < 0.05.

Wearing long-sleeve shirt and boots during mixing pesticides and long-sleeve shirt and long-sleeve trousers during spraying pesticides were excluded. The bold values mean significant results.

### Cognitive performance between pre-and post-pesticide application seasons

**Figure 3:**
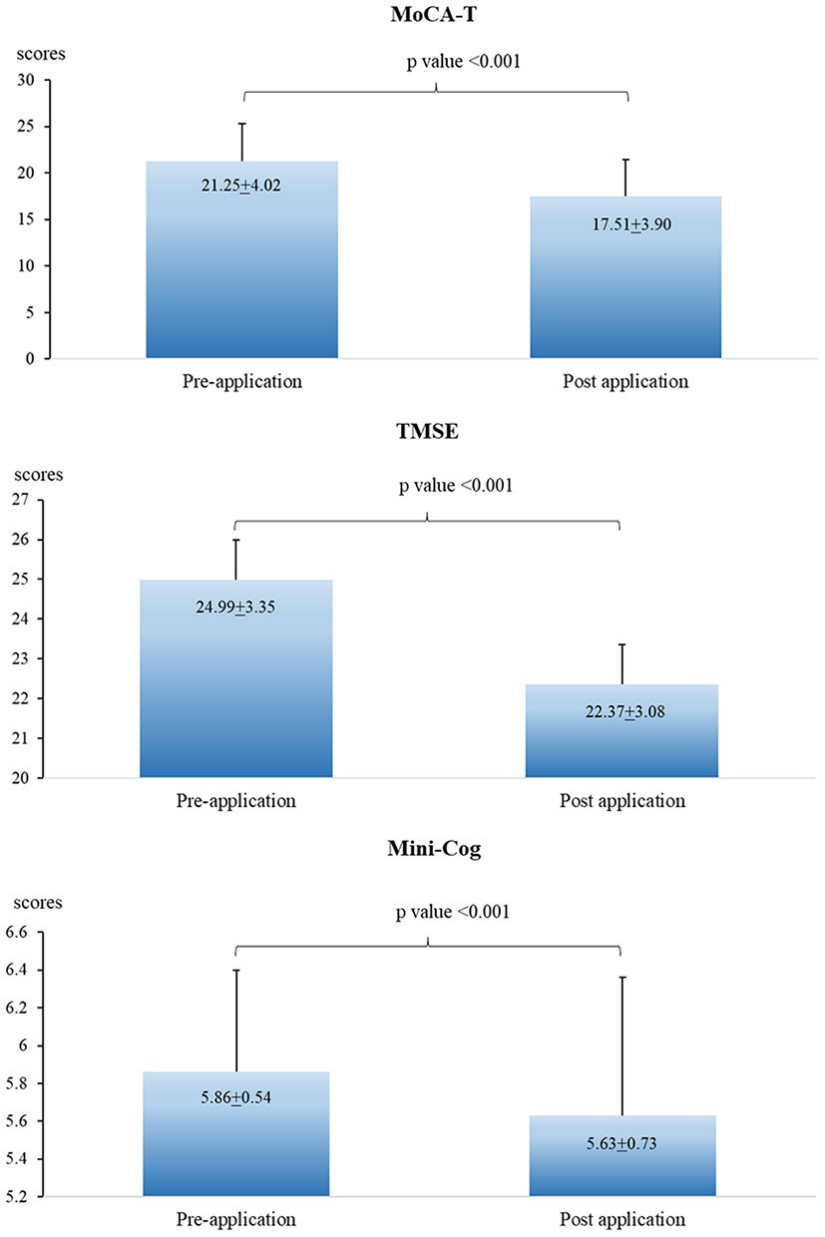
Cognitive decline between pre-and post-pesticide application seasons.

The scores of MoCA-T, TMSE, and Mini-cog in the post-pesticide application season were significantly lower than those in the pre-pesticide application season (Figure 3). However, no association was found when analyzing the association between urinary OP metabolite levels and cognitive performance (Table 5).

**Table 5:** Spearman rank correlation coefficient (*r*) between increased urinary OP metabolite levels and cognitive decline.

**Parameter**	**Increased total DEP**		**Increased total DMP**		**Increased total DAP**	
	***r***	***p* value**	***r***	***p* value**	***r***	***p* value**
Decreased MoCA-T	−0.021	0.854	−0.031	0.786	−0.062	0.588
Decreased TMSE	0.75	0.510	0.000	0.999	0.043	0.706
Decreased Mini-Cog	−0.124	0.277	−0.138	0.225	−0.139	0.222

MoCA-T, Montreal Cognitive Assessment-Thai; TMSE, Thai Mental State Examination; Mini-Cog, Mini cognitive test; r, Spearman rank correlation coefficient.

Total DEP, the summation of diethylphosphate (DEP), diethylthiophosphate (DETP), and diethyldithiophosphate (DEDTP).

Total DMP, the summation of dimethylphosphate (DMP), dimethylthiophosphate (DMTP), and dimethyldithiophosphate (DMDTP).

Total DAP, the summation of total DEP and total DMP.

## Discussion

Our findings show that the DEP metabolite was the major metabolite in urine samples as DEP had the highest detected and the highest levels when compared to other metabolites. It is possible that the DEP and DMP metabolites has different toxicokinetic characteristics. Diethyl OP are more lipophilic than dimethyl OP, therefore they can be stored in fatty tissues for longer periods of time, but dimethyl OP are immediately eliminated into the urine (26). Another possibility is that the most common OP used in this study area are triazophos (88.6%), followed by chlorpyrifos (15.2%). These two OP insecticides were classified as having diethyl groups; therefore, DEP metabolites were found as the major metabolite in urine samples.

A remarkable finding was that urinary OP metabolite levels post-pesticide application were significantly higher than those pre-pesticide application. Furthermore, the results also showed that the applicators who used motorized tank sprayers had higher total DEP levels than the applicators who used motorized knapsack sprayers. It is therefore likely that the type of spraying equipment was the primary factor influencing the risks of pesticide exposure. Hand and motorized knapsack sprayers had a maximum volume of 20 liters, and took about 1–2 h to empty whilst spraying pesticides on their field. On the other hand, motorized tank sprayers had a maximum capacity of 100 liters and took more than 2 h to spray the total contents onto the field. Therefore, the applicators who used motorized tank sprayers were more likely to be exposed to pesticides for longer periods of time than those who used other sprayers.

Improper use of PPE was also an important factor influencing pesticide exposure. Our findings revealed that the most regularly used PPE were long-sleeve shirt, and long-sleeve trousers, and boots, while goggles, gloves, and hat were worn the least frequently. The findings are consistent with a systematic review by Sapbamrer and Thammachai (27), which found that the most PPE worn among pesticide handlers in all world regions was long-sleeve shirt (66.1%), and long-sleeve trousers (71.1%), and the least common PPE worn were an apron (8.6%), goggles (24.3%), and gloves (40.5%). Theoretically, agricultural workers should wear all items of PPE to protect themselves from pesticide exposure *via* skin and inhalation while applying pesticides. However, with regard to protective clothing, agricultural workers should wear long-sleeve shirts and long-sleeve trousers made of waterproof cloth, according to the Department of Disease Control, Ministry of Public Health, Thailand (28). Although most Thai farmers always wore a long-sleeve shirt and long-sleeve trousers when applying pesticides, these PPE were usually made of woven fabrics. The main reasons for this are comfort and affordable price (15). According to Sapbamrer et al. (29), the efficiency of chlorpyrifos protection for woven clothing (76.5%) was much lower than shown by a Tychem^®^ coverall (90.7%). Also, our findings stated that goggles, gloves, and a hat were the least frequently used while applying pesticides. This could be because of a lack of practicality when working on their field. As a result, some chemicals from the pesticides can penetrate through the skin (19). A study by Aprea et al. (30) which investigated skin and respiratory exposure during spraying of lufenuron and re-entry in ornamental plant greenhouses suggested that the dose absorbed ranged from 0.144–0.171 and 0.005–0.124 μg/kg body weight during spraying and stapling, respectively, and the respiratory dose ranged from 68.7–74.6 and 0.022–0.636% of the total real dose during spraying and stapling, respectively. They also suggested that appropriate use of PPE and equipment was the fundamental aspect in reducing pesticide exposure.

Our findings also showed that increased urinary total DEP levels were associated with not wearing gloves while mixing pesticides and not wearing a mask while spraying pesticides, after adjusting for covariates. These results support the findings of Aprea et al. (5), who found that the highest levels of urinary OP metabolites in agricultural workers were linked to a lack of respiratory and hand protection. Measurement of urinary OP metabolites is used as a biomarker for monitoring of exposure to OP (31). As a result, the increased urinary OP metabolite levels may refer to the lack of PPE use during pesticide application. Agricultural workers had a risk of exposure to pesticides throughout any step of the pesticide application process, including mixing pesticides, spraying pesticides, and cleaning of sprayer equipment. Dermal contact and inhalation were the main routes of exposure (4). Agricultural workers can be exposed to pesticides by spills and splashes of concentrated and diluted pesticides in the mixing of the pesticides, therefore, not wearing gloves during this operation may increase the risk of being exposed to pesticide drips and splashes from both concentrated and diluted (4, 32, 33).

With regard to the spraying of pesticides, agricultural workers can be exposed to pesticides by direct contact with the spray. When pesticides are sprayed through nozzles, they produce very fine particles, therefore inhalation and skin contact are two main routes of exposure during this operation. Appropriate PPE, safety spraying equipment, and disposal methods for pesticide containers are important to reduce risks from pesticide exposure (34, 35). The recommendations by the Food and Agriculture Organization of the United Nations (FAO) and World Health Organization (WHO) (36) state that pesticide handlers should wear at least a respirator when handling pesticides. However, agricultural workers rarely comply with this recommendation. According to a systematic review by Sapbamrer and Thammachia (27), only 43.2% and 13.9% of pesticide handlers in all regions of the world wore masks and respirators, respectively, when working with pesticides. The main reasons given for non-compliance with the recommendations were thermal and mechanical discomfort, financial problems, and lack of availability (37). In Thailand, it was found that agricultural workers usually wore several types of masks made of woven fabrics, including cotton mask, robber mask, sun hat, and bandana. A study by Sapbamrer et al. (38) suggested that the insecticide filtration efficiency of respiratory protective equipment commonly worn in Thai farmers ranged from 64.9 to 95.4%, whereas half facepiece respirators were the most efficient in filtering insecticides, with a range of 96.5–98.9%. In the Sapbamrer study, the most common mask worn during spraying pesticides was a cloth mask (84.4%), followed by a surgical mask (13%), and an N95 (2.6%), respectively. As cloth and surgical masks have large pore sizes, they can only protect against large droplets, not against the fine mist used in the spraying of most pesticides. The surgical mask is designed for healthcare workers and infected people and some fine particles of OP can penetrate through the pores of these masks (36, 39). Subsequently, OP particles enter the respiratory system, accumulate in the body, and be excreted in urine in the form of OP metabolites.

Considering the effects of exposure to OP on cognitive performance, available previous studies report on evidence that exposure to pesticides and OP were associated with cognitive decline. Exposure to high levels of OP was significantly associated with the reduced scores of Mini-Mental State Exam (MMSE) (40–42). Exposure to high pesticide levels was also associated with the reduced MMSE and MoCA scores (13, 43, 44). In this study, the scores of MoCA-T, TMSE, and Mini-Cog test in post-pesticide application season were significantly lower than those pre-pesticide application season. However, no association was found between urinary OP metabolite levels and cognitive performance. It is possible that the cognitive decline found in this study might be due to other covariate factors. Another possibility is that exposure to OP at low levels and for short periods of exposure may not affect cognitive performance significantly. Table 6 presents average of urinary OP metabolite levels in other countries (45–51). Japanese farmers had the highest DAP levels (440.8 μg/g creatinine in summer), followed by floriculturists in Mexico (208.5 μg/g creatinine), and women farmworkers in South Africa (141.42 μg/g creatinine). However, pesticide applicators in Thailand (in the present study) had the lowest DAP levels (8.67 μg/g creatinine in summer). Therefore, long-term exposure and exposure to high levels of OP should be investigated further.

**Table 6:** Urinary OP metabolite levels (μg/g creatinine) in other countries.

**Country**	**Study population**	**Total DEP**	**Total DMP**	**Total DAP**	**Authors**
Thailand	Pesticide applicators (*n* = 79)^a^	4.43	2.22	8.67	Present study
South Korea	Male farmers (*n* = 104)^b^	10.32	4.07	14.39	Lee et al. (45)
USA (North Carolina)	Farmworkers (*n* = 203)^a^	6.9	18.09	24.99	Arcury et al. (46)
Peru	Pesticide applicators (n = 33)^a^	17.63	14.13	31.76	Yucra et al. (47)
Mexico	Flower growers (*n* = 117)^a, d^	18.95	81.17	102.71	Aguilar-Garduño et al. (48)
South Africa	Women farmworkers (*n* = 121)^c^	10.7	52.02	141.42	Motsoenen & Dalvie (49)
Mexico	Floriculturists (*n* = 104)^a^	33.6	121.5	208.5	Blanco-Mun~oz et al. (50)
Japan	Apple farmers (*n* = 147)^a^	5.8 (Summer)	43.2 (Summer)	440.8 (Summer)	Ueyama et al. (51)
		5.5 (Winter)	16.6 (Winter)	197.7 (Winter)	

^a^Presented with geometric mean (GM); ^b^presented with mean; ^c^presented with median; ^d^calculated from μmol/g creatinine to μg/g creatinine; DEP, diethylphosphate; DETP, diethylthiophosphate; DEDTP, diethyldithiophosphate; DMP, dimethylphosphate; DMTP, dimethylthiophosphate; DMDTP, dimethyldithiophosphate; DAP, dialkylphosphate; Total DEP, summation of DEP, DETP, and DEDTP; Total DMP, summation of DMP, DMTP, and DMDTP; Total DAP, summation of total DEP and total DMP.

The longitudinal follow-up design is a strength of this study as this study design can control invariant (person-specific) confounding factors. However, there are some limitations to this study. First, the small sample size would reduce statistical power and decrease the flexibility of the effect size. To overcome this in future studies a higher sample size would be used and also across different areas to increase the transferability of the findings. Secondly, other pesticides were also used in potato fields although OP were the most common insecticides employed in general use. We only measured urinary OP metabolites as a biomarker for exposure to OP, while other pesticides were assessed by a questionnaire. Measurement of other pesticides in urine or other biological fluids is therefore warranted in future studies.

## Conclusion

Our findings indicate that inappropriate PPE use during pesticide application was the major factor affecting urinary OP metabolite levels among pesticide applicators. Wearing gloves when mixing pesticides and wearing a mask when spraying pesticides could occupational exposure to pesticides. However, exposure to OP at low levels and for short periods of exposure may not be significant in causing neurological health problems. Long-term exposure and high levels of OP exposure should be investigated further. Continuous education and training regarding proper PPE use are vital in changing the behavior of agricultural workers toward PPE use.

## Data availability statement

The original contributions presented in the study are included in the article/Supplementary material, further inquiries can be directed to the corresponding author.

## Ethics statement

The studies involving human participants were reviewed and approved by the Research Ethics Committee, Faculty of Medicine, Chiang Mai University (no. 219/2021). The patients/participants provided their written informed consent to participate in this study.

## Author contributions

Conceptualization, methodology, and investigation: RS and JC. Validation, formal analysis, and resources: RS, JC, and SH. Data curation, writing original draft preparation, writing review and editing, supervision, project administration, and funding acquisition: RS. All authors have read and agreed to the published version of the manuscript.

## Funding

This study was supported by the Faculty of Medicine, Chiang Mai University (Grant Number 003/2565).

## Conflict of interest

The authors declare that the research was conducted in the absence of any commercial or financial relationships that could be construed as a potential conflict of interest.

## Publisher's note

All claims expressed in this article are solely those of the authors and do not necessarily represent those of their affiliated organizations, or those of the publisher, the editors and the reviewers. Any product that may be evaluated in this article, or claim that may be made by its manufacturer, is not guaranteed or endorsed by the publisher.
