# Supplementary material for: Exposure to organophosphate insecticides, inappropriate personal protective equipment use, and cognitive performance among pesticide applicators
Source: Front Public Health. 2022 Nov 17;10:1060284. doi: 10.3389/fpubh.2022.1060284 (PMC9712794; doi:10.3389/fpubh.2022.1060284)
Supplement: Supplementary file 1 [file Data_Sheet_1.docx]

**Appendix**

**Questionnaire and cognitive performance tests**

**--------------------------------------------------------------------------------**

**Questionnaire**

**Exposure to Organophosphate Insecticides, Inappropriate Personal Protective Equipment Use, and Cognitive Performance among Pesticide Applicators**

**Part 1. Socio-demographic characteristics and pesticide use data**

| Age ……(years) |  | | | | |
| --- | --- | --- | --- | --- | --- |
| Gender | 🞏Male 🞏Female | | | | |
| Marital status | 🞏Single/divorced 🞏Married | | | | |
| Education level | 🞏Primary school or lower 🞏Secondary school 🞏Bachelor’s degree or higher | | | | |
| Monthly income | 🞏≤10,000 Thai Baht | | | | |
|  | 🞏>10,000 Thai Baht | | | | |
| Underlying disease | | 🞏Yes 🞏No | | | |
| Body mass index (BMI)...........kg/m^2^ | | | | |  |
| Smoker of cigarettes | | | 🞏Yes 🞏No | | |
| Alcohol consumption | | | 🞏Yes 🞏No | | |
| Duration of pesticide use (years) | | | 🞏1-10 years | | |
|  | | | 🞏>10 years | | |
| Frequency of pesticide use | | | 🞏1-4 days/week | | |
|  | | | 🞏>4 days/week | | |
| Duration of pesticide spraying | | | 🞏0-2 hours 🞏>2 hours | | |
| Type of pesticide use | | | 🞏Triazophos 🞏Chlorpyrifos 🞏Diazinon | | |
|  | | | 🞏Glyphosate 🞏Paraquat  🞏Others (please specify)………………………… | | |
| Type of spraying equipment | | | 🞏Hand knapsack sprayer 🞏Motorized knapsack sprayer | | |
|  | | | 🞏Motorized tank sprayer | | |
| Type of spray nozzle | | | 🞏Low pressure 🞏High pressure | | |
| Storage of pesticide containers and equipment | | | | 🞏Home 🞏field | |
| Method of pesticide container disposal | | | | 🞏Landfill 🞏Burn 🞏Sell 🞏Reuse | |
| Distance from farm to residence | | | 🞏1-5 kilometers 🞏>5 kilometers | | |
| Have information about pesticides | | | 🞏Yes 🞏No | | |

**Part. 2 PPE use**

| **Wearing PPE during mixing pesticides** |  |
| --- | --- |
| Hat | 🞏Yes 🞏No |
| Goggles | 🞏Yes 🞏No |
| Boots | 🞏Yes 🞏No |
| Mask | 🞏Yes 🞏No |
| Gloves | 🞏Yes 🞏No |
| Long-sleeve shirt | 🞏Yes 🞏No |
| Long-sleeve trousers | 🞏Yes 🞏No |
| **Wearing PPE during spraying pesticides** |  |
| Hat | 🞏Yes 🞏No |
| Goggles | 🞏Yes 🞏No |
| Boots | 🞏Yes 🞏No |
| Mask | 🞏Yes 🞏No |
| Gloves | 🞏Yes 🞏No |
| Long-sleeve shirt | 🞏Yes 🞏No |
| Long-sleeve trousers | 🞏Yes 🞏No |
| **Wearing PPE during cleaning sprayer equipment** |  |
| Hat | 🞏Yes 🞏No |
| Goggles | 🞏Yes 🞏No |
| Boots | 🞏Yes 🞏No |
| Mask | 🞏Yes 🞏No |
| Gloves | 🞏Yes 🞏No |
| Long-sleeve shirt | 🞏Yes 🞏No |
| Long-sleeve trousers | 🞏Yes 🞏No |

**The Montreal Cognitive Assessment - Thai version (MoCA-T)**


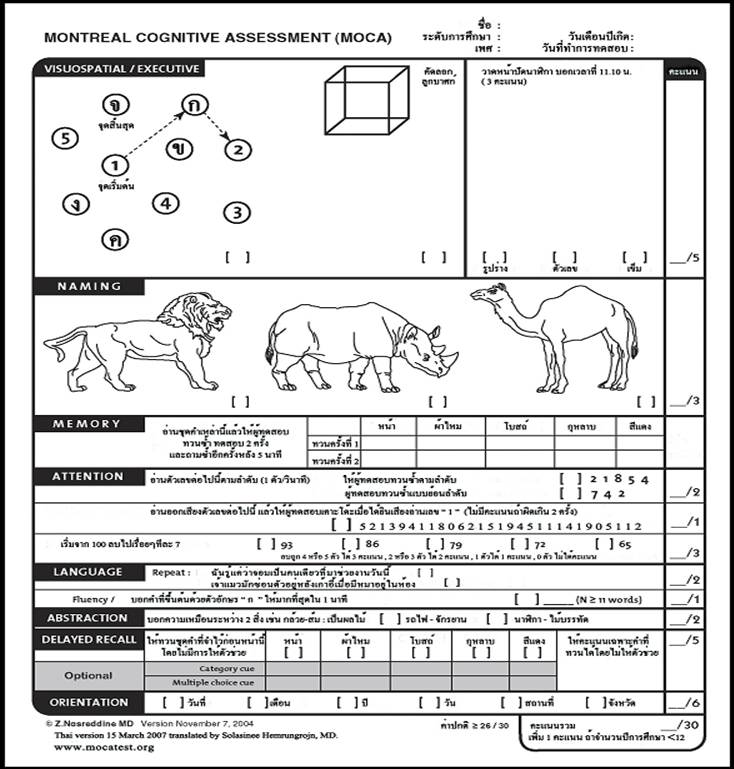


**The Thai Mental State Examination (TMSE)**

Code…………………………………………….

Examiner name…………………………………

Date of examination……………………….……

| **Questions** | **Maximum**  **points** | **points** |
| --- | --- | --- |
| **1.Orientation (6 points)** |  |  |
| 1.1 What is the Day? Date? Month? Time? | 4 |  |
| 1.2 Where are you now?  Street? Subdistrict? District? Province? Region? | 1 |  |
| 1.3 Who is he/she in a figure? | 1 |  |
| **2. Registration (3 points)**  The examiner names three unrelated objects slowly and then asks the patients/participants to name all three of them.  “River, Train, Pencil” | 3 |  |
| **3. Attention (5 points)**  Tell the date back  “Sunday, Saturday, Friday, Thursday, Wednesday, Tuesday, Monday” | 5 |  |
| **4. Calculation (3 points)**  Count backward from 100 by sevens, and keep going 3 times  “100, 93, 86, 79” | 3 |  |
| **5. Language (10 points)** |  |  |
| 5.1 What is this? (Clock, clothes,…) | 2 |  |
| 5.2 repeat the phase “Grandma took her grandson to buy snacks at the market” | 1 |  |
| 5.3 Follow what examiner told  “Pick up the paper with your right hand”  “Fold the paper in half”  “Hand the paper to the examiner” | 1  1  1 |  |
| 5.4 Read this sentence and do what it says  “Close your eyes.” | 1 |  |
| 5.6 Draw picture like this | 2 |  |
| 5.7 Answer what are similarity between banana and orange (fruit) | 1 |  |
| **6. Recall (3 points)**  Answer the name of three things that the examiner told you earlier in question no.2  (River, Train, Pencil) | 3 |  |
| **Total scores (30 points)** |  |  |

**Interpretation:** < 23 points : Dementia

≥ 23-30 points : Normal

**The Mini-Cognitive Test (Mini-Cog)-Thai version**

**Step 1. word registration**

Instruct the patient/participant listen carefully, and say that…

“I want you to remember these three words.”

“House, Ant, Red”

“Please say them for me now”

If the patient/participants can’t repeat these three words after three attempts, move to Step 2.

**-------------------------------------------**

**Step 2. Clock draw (2 points)**

The examiner said

“Please draw a clock on a paper”

“Start with draw a big circle”

“Please draw the hand of the clock to read a specific time at 11.10 o’clock”

If the patient/participants can’t draw within 3 minutes, move to Step 3.

**Scoring:**

🞏 2 points: Normal clock (correct sequence and positioning of all number in the clock with no missing and duplicate numbers. Correct two hands pointing, one pointing to the 11 and one pointing to 2. Hand length is not scored.)

🞏 0 point: Inability or refusal to draw

**-------------------------------------------**

**Step 3. Word recall (3 points)**

Ask the patient/participant to recall the words that the examiners stated in Step 1.

“Please let me now the words that I ask you to remember”

**Scoring:**

House 🞏 Correct answer (1 point) 🞏 Incorrect answer (0 score)

Ant 🞏 Correct answer (1 point) 🞏 Incorrect answer (0 score)

Red 🞏 Correct answer (1 point) 🞏 Incorrect answer (0 score)

**-------------------------------------------**

**Interpretation:** < 3 points: Possible abnormal cognitive function

3-5 points: Normal cognitive function
